# Immunohistochemical Predictors in Head and Neck Mucosal Melanoma

**DOI:** 10.1007/s12105-026-01969-1

**Published:** 2026-09-22

**Authors:** Sara C. Degerholm, Tuula A. Salo, Susanna Juteau, Matti O. Mauramo

**Affiliations:** 1 Department of Pathology, University of Helsinki and Helsinki University Hospital, Helsinki, Finland; 2 Department of Oral and Maxillofacial Diseases, Clinicum, Faculty of Medicine, University of Helsinki, Helsinki University Hospital, Helsinki, Finland; 3 Translational Immunology Research Program (TRIMM), University of Helsinki, Helsinki, Finland; 4 iCAN Digital Precision Cancer Medicine Flagship, University of Helsinki, Helsinki, Finland; 5 Research Unit of Population Health, Faculty of Medicine, University of Oulu, Oulu, Finland; 6 Medical Research Center, Oulu University Hospital, University of Oulu, Oulu, Finland

**Keywords:** Mucosal, Melanoma, Head and neck, Immunohistochemistry, Survival

## Abstract

**Background:**

Mucosal melanoma is a rare disease with a poor prognosis, and its clinical signs and symptoms are nonspecific. This study aims to identify histopathological features and immunohistochemical markers of head and neck mucosal melanoma that characterize the tumor immunophenotype and are associated with patient survival.

**Methods:**

The study analysed head and neck mucosal melanoma diagnosed at Helsinki University hospital between January 1, 2011, and June 8, 2023. Samples were obtained from the Helsinki Biobank in Finland. New slides were prepared and stained for hematoxylin and eosin (H&E), CD3, CD4, CD8, Granzyme B (GrzB), CD68, CD117, p16INK4a, p53, SOX10 and PRAME.

**Results:**

The study included 23 patients, 10 men and 13 women, aged 60–92 years (mean 75 years). The tumors were predominantly sinonasal. Ulceration was present in 15 cases, also 15 cases were amelanotic. Immunohistochemical staining SOX10 was positive in all tumors. PRAME was strongly positive in majority (20 cases, 87%). Strong p16 positivity was observed in 13 cases (57%), with a longer median survival compared to cases with weakened p16 expression (32.8 vs 13.8 months, *p* = 0.176). The median survival was also longer in the cases with high score of tumor infiltrating CD3-, CD8- and CD68-positive immune cells, though not statistically significant.

**Conclusions:**

Immunohistochemical markers routinely applied in cutaneous melanoma (SOX-10 and PRAME) are also expressed in head and neck mucosal melanoma. Tumour-infiltrating immune cells showed trend toward better survival, as has been previously found out in cutaneous melanoma. Strong p16 positivity may be an independent predictor of favorable prognosis in head and neck mucosal melanoma, irrespective of disease stage. Larger multicenter studies are warranted to validate these findings in this rare disease.

**Supplementary Information:**

The online version contains supplementary material available at https://doi.org/10.1007/s12105-026-01969-1.

## Introduction

The global incidence of cutaneous melanoma is increasing rapidly, with an estimated annual increase of 3–7% [1]. In Finland, cutaneous melanoma was the seventh most prevalent cancer in 2023, with incidences of 26.2 per 100,000 in women and 37.0 per 100,000 in men [2]. In contrast, mucosal melanoma is rare, constituting only 0.8–1.8% of all melanoma cases [3], with an annual incidence of 1.5 per million in the head and neck region [4]. Approximately 80% of head and neck mucosal melanomas involve the nasal cavity, septum or maxillary sinus [5]. The prognosis of mucosal melanoma is poor, and clinical signs and symptoms are often nonspecific. While mucosal melanoma differs biologically from cutaneous melanoma, treatment approaches remain similar due to the lack of clinical trials for mucosal melanomas [6].

In malignant melanomas there are a variety of cytomorphological features, architectural patterns and stromal changes that may be observed. The histopathological diagnosis of mucosal melanoma largely follows that of its cutaneous counterpart; however, Clark level of invasion and Breslow tumor thickness are not applicable, and the prognostic significance of ulceration remains unclear [7]. In addition, current American Joint Committee on Cancer (AJCC) TNM staging system and WHO Classification of Tumors classify all invasive head and neck mucosal melanomas as T3–T4 and stage III–IV, reflecting the inherently advanced stage at diagnosis [7, 8]. Common immunohistochemical markers in both mucosal and cutaneous melanoma include human melanoma black-45 (HMB-45), melanoma antigen A (Melan-A)/melanoma antigen recognized by T cells 1 (MART-1) and S-100 protein [9]. Additionally, SRY-related HMG-box-10 (SOX10) has been shown to be a sensitive marker of malignant melanoma [10]. The preferentially expressed antigen in melanoma (PRAME) is expressed not only in cutaneous melanoma, but also in mucosal melanoma, ocular melanoma, and various non-melanocytic malignant neoplasms [11]. The prevalence of BRAF mutations is notably lower in primary mucosal melanoma (5–11%) compared to cutaneous melanoma without chronic sun-induced damage (CSD) (59%). In contrast, cutaneous melanomas arising in chronically sun-damaged skin also show relatively low BRAF mutation rates (11%) and rarely harbour *BRAF* V600E mutations [6, 12]. Additionally, BRAF V600E occurs in more than 50% of low-CSD melanomas [7]. Considering BRAF subgroups, it has been proposed that non-V600 BRAF mutations are more common in mucosal melanoma than in cutaneous melanoma. In the study by Dumaz et al., BRAF mutations were found in 8% of mucosal melanomas, with 63% affecting the V600 codon and 37% involving other codons [13].

Several tumor suppressor genes are implicated in mucosal melanoma. The p53 gene is the most frequently mutated gene in human cancers, with wild-type p53 expression reported in 80–95% of cutaneous melanoma cases [14]. In contrast, p53 mutations occur in 9–29% of mucosal melanoma cases [15, 16]. It has been proposed that the role of p53 may be the transducer of keratinocytes in skin tanning and pigmentation and a promoter of melanocyte proliferation [17]. The p16INK4a, a cyclin-dependent kinase inhibitor, plays a significant role in melanoma and other cancers by inhibiting CDK4/6-mediated phosphorylation of the retinoblastoma tumor suppressor, leading to cell cycle arrest [18]. Mutations in cyclin-dependent kinase inhibitor 2a (*CDKN2a*) that encodes the tumor suppressor protein p16 are common in melanoma [19]. Additionally, loss of p16 expression is a consistent feature in cutaneous melanoma [20]. Mutations in the KIT gene which encodes the receptor tyrosine kinase, c-KIT (CD117), are also present in substantial subset of mucosal melanomas [21].

Understanding the interactions within the tumor stroma and microenvironment may help to differentiate aggressive tumors from those with a slower progression, potentially opening new opportunities to cancer treatment. The cancer cells may alter other cells and stroma to change in a way that it supports the cancer cell proliferation and migration. Assessment of tumor infiltrating immune cells is thought to be of critical importance in biomedical research and clinical pathology [22]. In cutaneous melanoma, tumor-infiltrating lymphocytes (TILs) serve as a positive prognostic factor and represents varying lymphocyte subsets [23]. In deeply invasive pT4 cutaneous melanomas compared with superficial pT1 melanomas, tumor cells exhibit stronger immunohistochemical staining for CD8 (cytotoxic T lymphocytes) and granzyme B (GrB), indicating heightened cytolytic activity. A low CD8/GrB ratio is associated with better recurrence-free survival in primary cutaneous melanomas [24]. The tumor microenvironment in melanoma shows a close association between intratumoral CD8+ cells and PDL1 expression, which is more prevalent on macrophages than in melanoma cells [25]. A meta-analysis confirms the favourable prognostic role of the CD3+, CD4+, CD8+, FOXP3+, and CD20+ TILs in the overall survival of melanoma patients, with studies focusing on primary cutaneous melanoma [26].

The aim of this study is to identify histological parameters and immunohistochemical markers of head and neck mucosal melanoma that are related to patient survival. The hypothesis is that the immunohistochemical stainings commonly used in cutaneous melanoma are also reliable in head and neck mucosal melanoma. Additionally, an aim is to investigate whether these markers can help predict tumor aggressiveness and to characterize features of the tumor microenvironment that are associated with prognosis. Improved diagnostics may enable more individualized treatment in future and help differentiate malignancies with an aggressive course.

## Materials and Methods

The study included malignant mucosal melanoma cases from the head and neck diagnosed in Helsinki University hospital between January 1, 2011, and June 8, 2023. The patients included in this study were also included in our previously published 56-patient Helsinki pseudonymized register-based cohort focusing on the incidence and clinical presentation of head and neck mucosal melanoma. The present tissue-based histopathological and immunohistochemical analyses were prepared for this study and are not reported in the previous publication [4]. This study was performed in line with the principles of the Declaration of Helsinki. Approval was granted by the Ethics Committee of the Northern Ostrobothnia Hospital District PPSHP EETTMK: 101/2020 § 20 and Helsinki University Hospital: HUS/150/2022 § 25, HUS/124/023 § 21.

Samples were collected from the Helsinki Biobank, Helsinki, Finland. Only primary malignant mucosal melanomas were included; melanoma in situ and cutaneous melanoma were excluded. Three cases were excluded due to lack of the patient consent for use of samples from the Helsinki Biobank. Clinical follow-up was updated until May 5, 2025, which served as the data cut-off. Overall survival was calculated from the date of the sample to death from any cause, and patients alive at the data cut-off were censored. Nineteen patients died during follow-up, with survival times ranging from 4 to 81 months; the remaining four patients were censored alive at 24, 41, 166, and 173 months. Data on age, sex, time of death, stage and pathology reports—including date, tumor anatomic site, previous immunohistochemical stainings and genetic mutations—were collected from patient records.

All the samples were re-evaluated by two independent pathologist (SD, MM) to confirm the diagnosis of melanoma and eligibility for the study. New slides were prepared from most representative formalin-fixed, paraffin-embedded tissue blocks and stained for H&E, CD3 (universal T cell marker), CD4, CD8, GrzB, CD68 (histiocytic marker), CD117, p16INK4a, p53, SOX10 and PRAME. Staining was performed using Ventana Benchmark Ultra Plus (Roche/Ventana, Tucson, AZ, USA) instrument with CC1 high pH pretreatment buffer (Roche/Ventana, 950–900). The polymer- based 3-step detection kit, the Optiview DAB IHC (Roche/Ventana, 6,396,500,001) was used for antibodies CD4, CD117, GRB, SOX10 and p16. The UltraView Universal DAB detection kit (Roche/Ventana, 05269806001) was used for the antibodies CD3, CD8, CD68 and p53. The UltraView Universal Alkaline Phosphatase Red detection kit (Roche/Ventana, 05269814001) was used for PRAME. Detailed information regarding the antibodies is included in Supplementary Table 1.

Stainings were evaluated by two independent pathologist (SD, MM). The PRAME expression was evaluated by the percentage of tumor cells with positive nuclear staining. The expression was scored on a 0 to 4 scale: 0: <1%, 1: 1–25%, 2: 26–50%, 3: 51–75%, 4: >76% positive cells as presented by Lezcano et al. [27].

The percentage of CD117-positive cells was estimated, and cases were classified as 0 (no positive cells), <10%, 10–50%, or >50% positive cells, as described by Radu et al. [28]. The p53 pattern was classified as wild-type when fewer than 50% of tumor cells showing nuclear staining, clonal when ≥50% were positive, and null (complete loss of staining with preserved internal controls) [29].

**Fig. 1:**
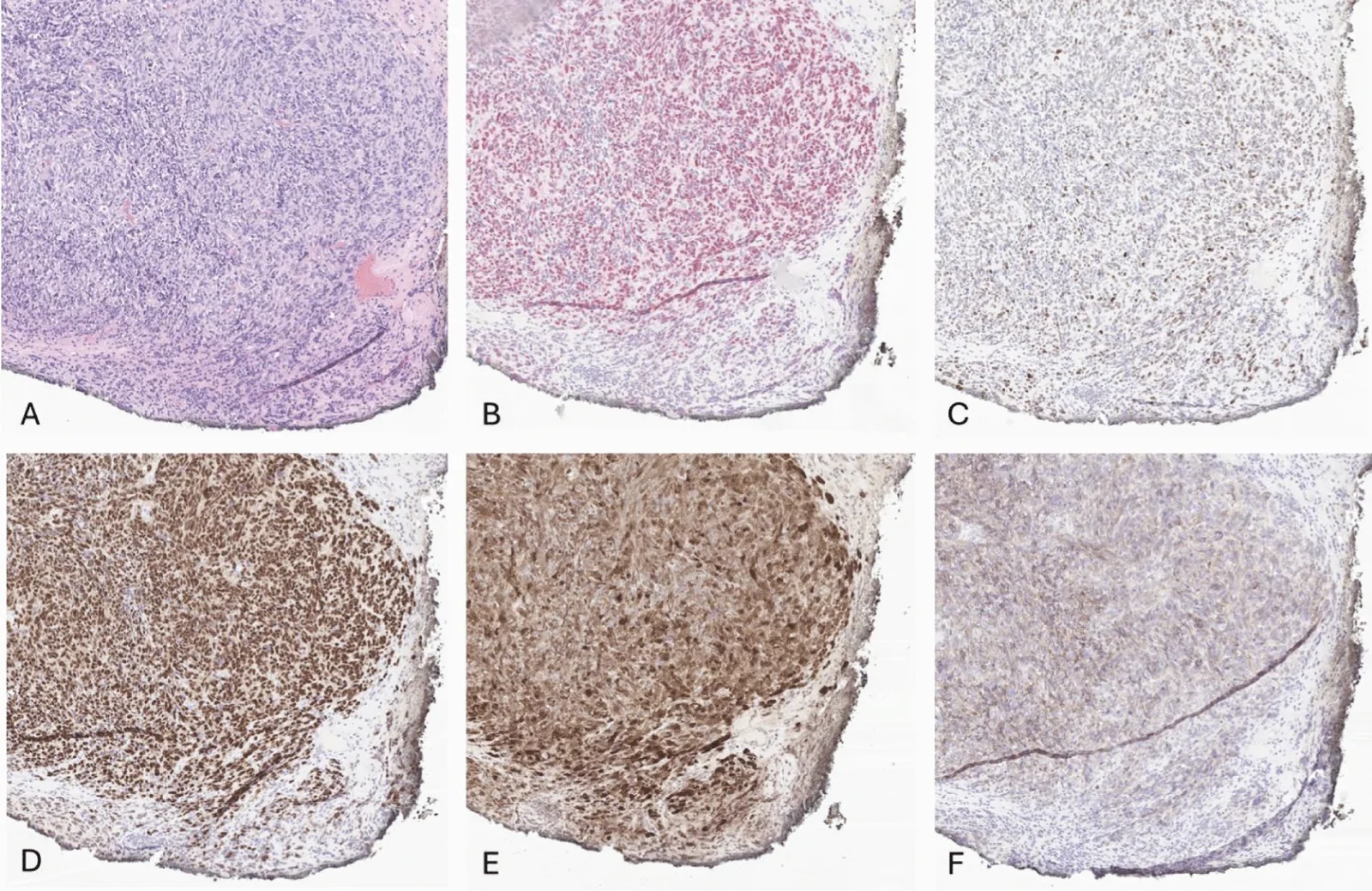
Immunohistochemical staining patterns in Head and Neck Mucosal Melanoma (HNMM). **a** H&E. **b** PRAME positivity in melanoma cells. **c** p53 wild type staining pattern. **d** SOX-10 positivity. **e** Strong positive p16 staining pattern. **f** Partial CD117 expression of melanoma cells. (10× magnification)

p16 expression was evaluated semiquantitatively using modified criteria based on Tanaka et al. [30]. Positive staining was defined as nuclear staining in melanoma cells that was stronger than cytoplasmic staining and stronger than staining in tumor stromal cells. Each tumor was assigned to one of the following groups according to the staining ratio and pattern of melanoma cells: 0 (0%), 1 (1–10%), 2 (11–30%), 3 (> 31%, partial staining with complete negative areas), and 4 (at least focal uniform, block-positive p16 staining combined with at least heterogeneous, checkerboard-like staining throughout the tumor). p16 staining was classified as strongly positive when the tumor showed a group 4 staining pattern, and as weakened when staining was assigned to groups 0–3 (Fig. 1).

**Fig. 2:**
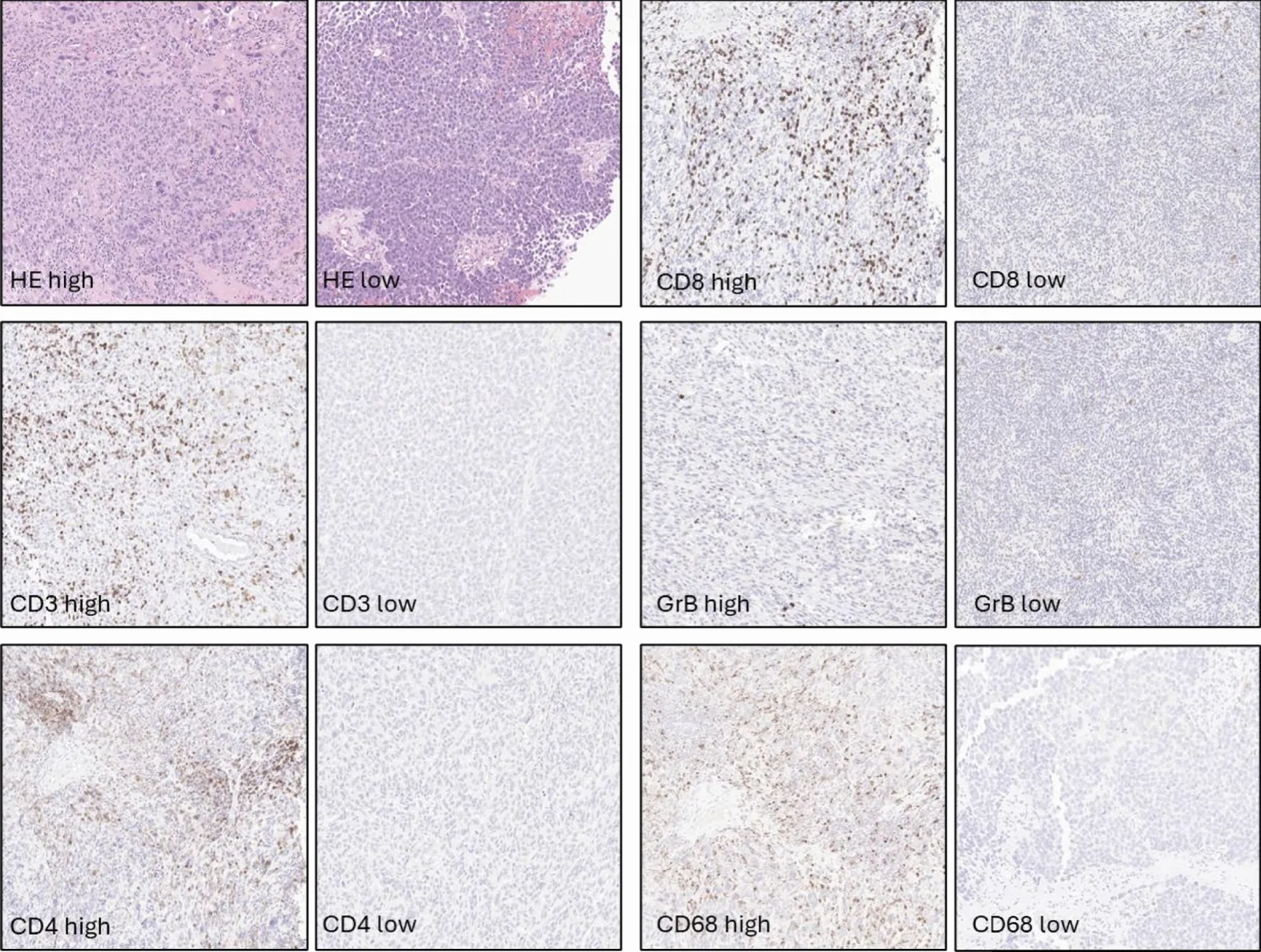
Tumor infiltrating cells in Head and Neck Mucosal Melanoma (HNMM). Representative images show HNMM samples with low and high infiltration of tumour infiltrating lymphocytes (TIL) in HE along with CD3, CD4, CD8, GrB and CD68 positive cells. (10× magnification)

The lymphocyte score was defined according to the scoring system presented by Ledderose et al. [31]. For each sample, the lymphocyte distribution score (percentage of tumor area infiltrated by lymphocytes) and the lymphocyte density score (intensity within the infiltrate) were calculated, total sum score ranging from 0 to 6. Scores from 0 to 4 were classified as low, while scores from 5 to 6 were classified as high. The lymphocyte distribution score ranged from 0 to 3 and was defined as follows: 0 = absence of lymphocytes within the tumor; 1 = lymphocytes occupying < 25% of the tumor tissue; 2 = lymphocytes occupying 25–50% of the tumor tissue; and 3 = lymphocytes occupying > 50% of tumor tissue. Lymphocyte density score ranged from 0 to 3 and was defined as follows: 0 = absent, 1 = mild, 2 = moderate, and 3 = severe (Fig. 2). Peritumoral positive cells were defined as cells distributed along the stromal-tumor interface at tumor edge within 1 mm, as prescribed by Yusa and Sabbatino [32, 33].

Statistical analysis was performed using SPSS Statistics program 29.0.0.0. (241) (IBM Corporation, Armonk, New York, NY, USA). Descriptive univariate analyses were conducted by sex, ulceration, pigment, immunohistochemical staining patterns and survival status (alive vs. deceased). Survival was analyzed using Kaplan–Meier estimates and compared with the log-rank test, with a *p*-value < 0.05 considered significant. The association between p16 expression and overall survival was further evaluated using Cox proportional hazards regression, adjusting for stage.

## Results

The study included 23 patients, from which 10 were men and 13 women, diagnosed over a 12-year period. The mean age was 75 years (range: 60–92 years). At the data cut-off, 19 of 23 patients had died and four patients were alive and censored. The median overall survival was 22.3 months. No significant difference in survival was observed between sexes: men 22.3 months vs women 20.4 months, *p* = 0.776. The primary tumors were predominantly sinonasal, with 21 cases (91.3%). There were also one oral and one laryngeal tumor. According to the AJCC TNM staging system, all invasive head and neck mucosal melanomas are categorised as T3–T4 and stage III–IV [8]. In this study, most cases were stage III (15 cases, 65%), with the remainder stage IV (8 cases, 35%). As expected, survival was longer in stage III patients than in stage IV patients (median 24.6 vs 12.0 months), although the difference was not statistically significant (*p* > 0.05), (Table 1). Most patients (74%) received postoperative radiotherapy as adjuvant treatment for locoregional control. One patient received chemoradiotherapy; otherwise, no systemic therapy was administered postoperatively. Systemic immunotherapy was initiated later in six patients after progression to metastatic disease, and chemotherapy was given to two patients. A multivariable analysis adjusting for treatment was not possible due to the small sample size.

**Table 1:** Patient and tumor characteristics in relation to survival

	n (%)	Deaths, n (%)	Median survival (mo)	*p*-value*	95% CI	
					Lower	Upper
Total	23 (100)	19 (83)	22.3	N/A	15.570	29.030
Men	10 (43)	8 (80)	22.3	0.776	15.637	28.963
Women	13 (57)	11 (85)	20.4		0.000	54.859
Stage						
III	15 (65)	12 (80)	24.6	0.234	9.651	39.549
IV	8 (35)	7 (88)	12.0		0.000	26.552
Ulceration						
Positive	15 (71)	13 (87)	22.3	0.408	11.065	33.535
Negative	6 (29)	4 (67)	33.2		0.000	94.653
Pigment						
Positive	8 (35)	6 (75)	20.3	0.556	1.878	47.322
Negative	15 (65)	13 (87)	24.6		11.153	29.447

Abbreviations: n=number of cases, mo=months, CI=confidence interval, *Log Rank (Mantel-Cox)

KIT mutations (exons 9,11,13,17) were previously studied in 17 of the 23 cases, with no KIT mutations detected. The mutation profile covering ALK, EGFR, KEAP1, KIT, KRAS, MET, NRAS, PDGFRA, PIK3CA, STK11 and BRAF (exons 11–15) was previously examined in 13 cases, in which two NRAS and one KRAS mutation were detected. No BRAF mutations were found.

Histologically, ulceration was present in 15 cases (71.4%) but showed no significant association with survival, although median survival was slightly longer in cases without ulceration (33.2 vs 22.3 months). In two cases the ulceration could not be evaluated due lack of the adjacent epithelia. Pigment was found in 8 cases (34.8%), while 15 cases (65.2%) were amelanotic. The mitotic rate varied from 0 to 38 mitoses/mm^2^ (mean: 12). None of these histological characteristics were statistically significantly related to survival (all *p*-values > 0.05) (Table 1).

SOX10 was positive in all tumors (23 cases, 100%). PRAME immunohistochemistry was strongly positive (score 4) in 20 cases (87%), with the remainder showing low positivity. p53 was wild type in 19 cases (86%), while one case (5%) was aberrantly positive and two cases (9%) aberrantly negative. In one case p53 staining was not reliable due the strong pigmentation of the tumor that affected the evaluation. The survival was better in patients with aberrant p53 (median 32.8 vs 20.4 months, respectively, *p* > 0.05). CD117 was low or negative in 9 cases (41%), while 13 cases (59%) expressed positivity, however association with survival was quite low (median 24.6 vs 20.4 months, *p* > 0.05) and there was no association with previously examined KIT-mutation profile. In one case CD117 staining was not reliable due the strong pigmentation of the tumor that affected the evaluation.

**Fig. 3:**
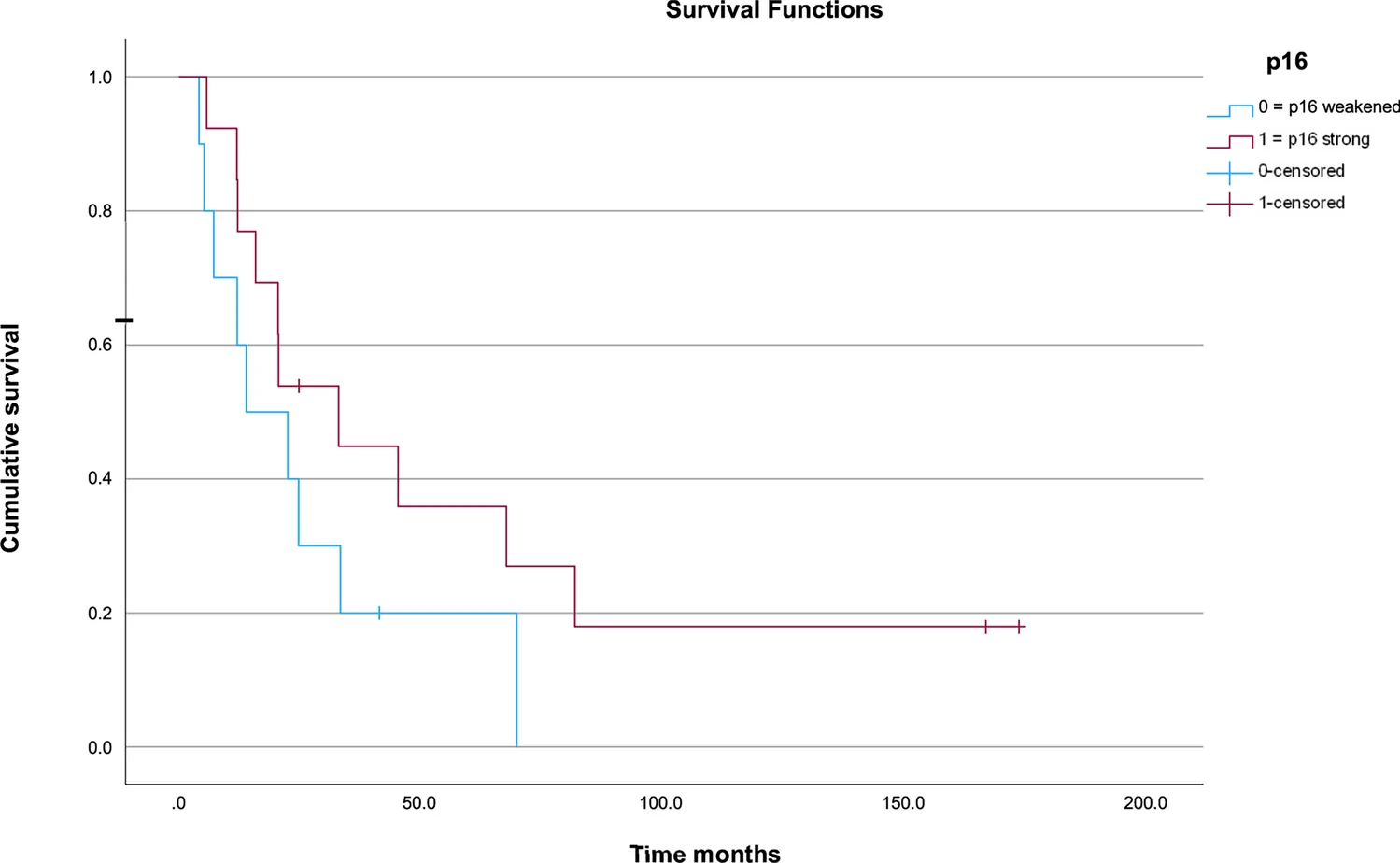
Kaplan–Meier estimates for p16 staining and survival

Strong p16 positivity was observed in 13 cases (57%), with a longer median survival compared to cases with weakened p16 expression (32.8 vs 13.8, *p* = 0.176), (Fig. 3). It was also found that the proportion of cases with weakened p16 staining was considerably higher in stage IV than in stage III (63 vs 33%). After adjustment for stage in Cox regression analysis, strong p16 expression was associated with a lower hazard of death compared with weakened p16 expression, although the association was not statistically significant (HR 0.60, 95% CI 0.22–1.64).

In most cases CD3, CD8 and CD68 positive tumor-infiltrating immune cells resulting high score of 5 or 6 were observed (n=20/19/19 and 87%/83%/83%, respectively). The median survival was longer in the cases with high positivity in tumor infiltrating cells: 20.4 months (CD3), 22.3 months (CD8), 24.6 months (CD68), compared to cases with low scores (12.0^#^/12.0/11.8 months respectively), although statistical significance was not obtained (Table 2). Half of the cases (n = 11, 48%) achieved high score for GrB, but this finding was not associated with survival. High score CD4 positivity was identified in 11 cases (48%), with low score in 12 cases (52%), with no association with median survival (22.3 vs 20.4 months).

**Table 2:** Results in immunohistochemical stainings in relation to survival

IHC	n (%)	Deaths, n (%)	Median survival (month)	*p*-value*	95% CI	
					Lower	Upper
Tumor supressors						
p53ab	3 (14)	3 (100)	32.8	0.905	0.000	66.407
p53wt	19 (86)	16 (84)	20.4		11.015	29.785
CD117+	13 (59)	11 (85)	20.4	0.556	10.418	30.382
CD117−	9 (41)	8 (89)	24.6		12.036	37.164
p16 strong	13 (57)	10 (77)	32.8	0.176	12.018	53.582
p16 weakened	10 (43)	9 (90)	13.8		0.000	29.915
Tumor infiltrating immune cells						
CD3+	20 (87)	18 (90)	20.4	0.149	16.017	24.783
CD3−	3 (13)	1 (33)	12.0^#^		N/A	N/A
CD4+	11 (48)	10 (91)	22.3	0.591	2.979	41.621
CD4−	12 (52)	9 (75)	20.4		13.101	27.699
CD8+	19 (83)	17 (89)	22.3	0.339	16.186	28.414
CD8−	4 (17)	2 (50)	12.0		N/A	N/A
GZB+	11 (48)	9 (82)	22.3	0.876	0.000	53.473
GZB−	12 (52)	10 (83)	20.4		13.101	27.699
CD68+	19 (83)	16 (84)	24.6	0.852	7.673	41.527
CD68−	4 (17)	3 (75)	11.8		5.528	18.072

IHC = immunohistochemical staining, n = number of cases, mo = months, CI = confidence interval, *Log Rank (Mantel-Cox), ^#^n = 1

In five cases the peritumoral area could not be reliably evaluated. In all evaluable cases (n = 18), moderate or strong peritumoral positivity for CD3, CD4, and CD8 was observed. Moderate to strong peritumoral positivity in CD68 was found in 15 cases (83%). GrzB was absent or mild in 4 cases (22%), with survival of 11.9 months compared to 22.3 months in the GrzB positive group. Due the small sample size, statistical significance remains limited.

## Discussion

This study aimed to identify histopathological features and immunohistochemical profile of mucosal melanoma of the head and neck in relation to patient prognosis. The findings indicate that immunohistochemical stainings commonly used in cutaneous melanoma, including SOX10 and PRAME, are also frequently expressed in mucosal melanoma. Tumor-infiltrating immune cells were associated with improved survival, suggesting their potential prognostic value. It was found that the cases with weakened p16 staining had shorter median survival than those with strongly positive staining. In addition, the higher proportion of cases with weakened p16 expression in advanced disease (stage IV 63% vs stage III 33%) highlights the reduction of p16 expression as the tumor progresses. The better prognosis associated with p16 positivity persisted even after adjusting for disease stage. Classical histological features, such as ulceration and pigment status, were not statistically significantly associated with survival.

### Demographics of the Patients

In this study, there were 23 cases of head and neck mucosal melanoma diagnosed over period of 12 years, with the mean patient age of 75 years, ranging from 60 to 92. The age of the patients was slightly older than in previous studies [6, 34]. The proportion of women was higher (57%), consistent with previous studies [6, 35]. There has been discussion whether this is because of the melanoma in vulvovaginal area, though it can’t explain the difference in this cohort [35]. There was no difference in survival between sexes, echoing inconsistent results from other studies [36].

### Histology of Mucosal Melanoma

Consistent with previous studies, most cases were ulcerated (71%) [27, 28]; however, ulceration was not significantly associated with survival in this cohort, although there was a trend toward longer survival in patients without ulceration. This finding may support some earlier reports identifying ulceration as an independent predictor of survival in head and neck mucosal melanoma [27]. The prognostic significance of ulceration in mucosal melanoma remains uncertain and warrants further investigation. Pigment was found in only 35% of cases, with 65% being amelanotic. It is known that mucosal melanoma can be amelanotic and in some studies it is associated with worse prognosis [6]. This might be because of the clinical difficulties in diagnosing amelanotic melanoma. This study found no significant survival difference based on pigment status (*p* = 0.556).

In immunohistochemical stainings, all cases were positive for SOX10, consistent with findings in cutaneous melanoma and suggested also for mucosal melanoma [8, 30]. PRAME immunohistochemistry was strongly positive in 87% of cases, with the remainder showing weak positivity. These results suggests the utility of SOX10 and PRAME as diagnostic markers in mucosal melanoma and comparing these stainings with diagnostic mimics would be beneficial in future studies [9, 31].

### Tumor Suppressors

Given that p53 is the most frequently mutated gene in human cancers but typically shows wild-type expression in cutaneous melanoma, it is relevant to assess its staining pattern in head and neck mucosal melanoma. The rate of aberrant p53 staining in this study (14%) aligns with previous reports [12, 13]. No statistically significant survival differences were observed based on p53 staining.

Another tumor suppressor gene, KIT, is more frequently mutated in mucosal melanoma than in cutaneous melanoma (15 vs 3,7%) [37]. In this study, 74% of cases had been previously tested for KIT mutations, with none detected. It has been reported that CD117 expression, which identifies the KIT protein, decreases as tumors progress [33]. Here, CD117 staining was positive in 59% of cases, while the remaining cases showed lower expression. The staining profile was not associated with the survival or previously examined mutational status.

Furthermore, changes in the CDKN2A tumor suppressor gene, which encodes the p16 protein, are common drivers of melanoma progression in cutaneous melanoma [34]. Reduced p16 expression has been linked to poorer outcomes [35], and the loss of p16 immunohistochemical expression is more prevalent in cutaneous than mucosal melanoma [34, 36]. In this study, the weakened p16 staining was more frequently observed in advanced-stage cases (stage IV 63% vs stage III 33%), and cases with weakened p16 staining had a shorter median survival than those with positive staining (13.8 vs 32.8 months); however, these differences did not reach statistical significance, probably because of the small cohort (*p* > 0.05). In Cox regression analysis, the better prognosis associated with p16 positivity persisted even after adjusting for disease stage. As CDKN2A gene analysis was not conducted, its relationship to p16 immunohistochemical expression could not be evaluated.

The results show a strong but not statistically significant trend indicating that strong p16 positivity is an independent marker of favorable prognosis, regardless of disease stage, in head and neck mucosal melanoma. Although the statistical evidence is insufficient for definitive conclusions, the finding is clinically meaningful and suggestive and warrants further studies.

### Tumor Microenvironment

In cutaneous melanoma, the presence of TILs is regarded as a prognostic factor [22]. The role of tumor-infiltrating immune cells in mucosal melanoma has yet to be established, although some lymphocyte subgroups have been linked to survival [31]. In this study, CD3, CD8 and CD68 positive tumor infiltrating immune cells were observed in most tumors. While not statistically significant, the high score positivity of tumor-infiltrating immune cells was associated with better survival with across all subtypes. Regarding peritumoral lymphocytes, no significant differences were found between subgroups.

## Conclusions

This study confirms that immunohistochemical stainings used in cutaneous melanoma are also expressed in head and neck mucosal melanoma. The findings highlight the independent prognostic significance of tumor-infiltrating immune cells in head and neck mucosal melanoma, although obtaining statistical significance in a single-centre study is challenging due the rarity of the disease. Our findings suggest that strong p16 positivity may be an independent predictor of favorable prognosis in head and neck mucosal melanoma, irrespective of disease stage. Future larger multicentre studies could strengthen these results and yield additional information of specific subtypes in head and neck melanoma.

## Supplementary Information

Below is the link to the electronic supplementary material.

Supplementary file1 (DOCX 13 kb)

## Data Availability

The datasets generated and analysed during the current study are not publicly available due to privacy ant ethical reasons but are available from the corresponding author on reasonable request.
