# Supplementary material for: Immunohistochemical Predictors in Head and Neck Mucosal Melanoma
Source: Head Neck Pathol. 2026 Sep 22;20(1):99. doi: 10.1007/s12105-026-01969-1 (PMC13597977; doi:10.1007/s12105-026-01969-1)
Supplement: Supplementary file 1 [file 12105_2026_1969_MOESM1_ESM.docx]

Supplementary Table 1: Detailed information for the used antibodies.

| Staining | Clone | Manufacturer | Item number | Dilution |
| --- | --- | --- | --- | --- |
| CD3 | 2GV6 | Ventana, Roche | 790-4341 | RTU |
| CD4 | SP35 | Cell Marque | 104R-16 | 1:100 |
| CD8 | 4B11 | Novocastra | NCL-L-CD8-4B11 | 1:50 |
| CD117 | EP10 | Ventana, Roche | 790-7061 | RTU |
| CD68 | KP1 | Dako, Agilent | M 0814 | 1:1000 |
| GRB | GrB-7 | Monosan | MON 7029C | 1:40 |
| SOX10 | SP267 | Ventana, Roche | 760-4968 | RTU |
| PRAME | RBT-PRAME | Bio SB | BSB-2374-1 | 1:100 |
| P16 (p16INK4a) | E6H4 | Ventana, Roche | 852-4713 | RTU |
| P53 | DO-7 | Dako, Agilent | M 7001 | 1:500 |
